# Association between early-phase opioid use and outcomes in extremely preterm infants: A nationwide study

**DOI:** 10.1038/s41390-025-03998-y

**Published:** 2025-03-18

**Authors:** Hiroki Kitaoka, Takaaki Konishi, Yoshihiko Shitara, Atsushi Ito, Kohei Kashima, Yuya Kimura, Hiroki Matsui, Motohiro Kato, Naoto Takahashi, Hideo Yasunaga

**Affiliations:** 1 Department of Pediatrics, The University of Tokyo Hospital, Tokyo, Japan; 2 Department of Neonatology, Tokyo Women’s Medical University Hospital, Tokyo, Japan; 3 Department of Clinical Epidemiology and Health Economics, School of Public Health, The University of Tokyo, Tokyo, Japan

## Abstract

**Background:**

Opioids are often administered for extremely preterm infants to prevent morbidities (e.g., intraventricular hemorrhage); however, their short-term outcome remains unclear. We aimed to assess the association between early-phase opioid use and in-hospital outcomes in extremely preterm infants.

**Methods:**

This retrospective nationwide cohort study analyzed data from the Diagnosis Procedure Combination database in Japan. A total of 18,794 extremely preterm infants hospitalized between July 2010 and March 2021 were included. The patients were divided into those who received early-phase opioids (*n* = 4806) and those who did not (*n* = 13,988). We performed a 1:2 propensity score-matched analysis adjusting for patient backgrounds.

**Results:**

In-hospital mortality, intraventricular hemorrhage, and periventricular leukomalacia occurred in 8.5%, 13%, and 2.1% of the patients, respectively. The incidences of in-hospital mortality (12% vs. 12%) and intraventricular hemorrhage (14% vs. 15%) did not differ between the two groups after 1:2 propensity score matching. The patients who received early-phase opioids had a lower incidence of periventricular leukomalacia than those who did not (1.7% vs. 2.2%). However, bronchopulmonary dysplasia occurrence (65% vs. 58%), home assistive technology use (19% vs. 15%), and length of hospital stay (125 vs. 122 days) were increased for patients who received early-phase opioids.

**Conclusion:**

In this large retrospective study, early-phase opioid use in extremely preterm infants was not associated with increased mortality or intraventricular hemorrhage. Periventricular leukomalacia slightly decreased.

**Impact:**

- This large retrospective nationwide study of 13,988 extremely preterm infants revealed that using early-phase opioids was not significantly associated with in-hospital mortality or intraventricular hemorrhage but was significantly associated with a decrease in periventricular leukomalacia.
- Early-phase opioids may not increase mortality or intraventricular hemorrhage, in contrast to the results of previous studies. Thus, these results could help clinicians select opioids as sedative agents for extremely preterm infants.

## Introduction

Extremely preterm infants under 28 weeks of gestation often undergo invasive procedures, such as blood sampling, central venous catheterization, and intubation.^1,2^ These invasive procedures potentially cause severe complications, including intraventricular hemorrhage (IVH) and periventricular leukomalacia (PVL).^3^ To reduce severe complications in preterm infants, sedation and analgesic drugs such as opioids, midazolam, and phenobarbital are administered,^1,4^ which are essential because IVH and PVL can cause neurodevelopmental impairment.^1,3–5^ Thus, opioids (e.g., fentanyl and morphine) have been used in preterm infants in the neonatal intensive care unit (NICU).^6^

Previous research on early-phase opioid use in extremely preterm infants is limited due to insufficient adjustments for confounding factors. A retrospective study of 4781 such infants found increased in-hospital mortality, IVH, PVL, bronchopulmonary dysplasia (BPD), and retinopathy of prematurity associated with early-phase opioid use without adjusting for crucial confounding factors, such as drug use and procedures.^7^ Other small previous studies have linked early-phase opioid use with unfavorable mortality and morbidities without adjusting for background factors.^8,9^ Moreover, multiple randomized controlled trials and meta-analyses have not confirmed the effectiveness of early-phase opioid use in preterm infants (including extremely preterm infants) due to small sample sizes.^10–17^ These short-term outcomes affect the life of infants and are often associated with long-term outcomes such as neurodevelopmental impairment.

Given the high prevalence of preterm birth in high-income countries,^18^ investigating the benefits of early-phase opioid use is necessary. Investigation of the short-term outcomes of early-phase opioid use is important to improve the treatment in NICUs. Therefore, we aimed to investigate the association between early-phase opioid use and outcomes in extremely preterm infants after adjusting for potential confounders using a nationwide database in Japan.

## Methods

### Database

This nationwide retrospective study used the Diagnostic Procedure Combination database. This database contains administrative claims data and discharge abstracts of 8,000,000 inpatients per year from > 1200 hospitals in Japan.^19^ The database covers approximately 90% of hospitals with NICUs.^20^ The participation of community hospitals was voluntary although all 82 university hospitals in Japan were required to join the database. The requirement for informed consent was waived due to the anonymity of the database. The Institutional Review Board of the University of Tokyo approved this study (approval number: 3501-(5) [May 19th, 2021]).

The database includes the following data: sex of the patient, gestational age; birth weight; age at admission; main diagnoses; comorbidities at admission; and complications after admission recorded in Japanese text and using the *International Classification of Disease, Tenth Revision* (ICD-10) codes indexed by the original Japanese codes; patients’ transportation from other hospitals or in-hospital births; length of stay; discharge status; and hospitalization costs. Hospitalization costs were calculated based on the reference prices in the Japanese national fee schedule for item-by-item prices for surgical, pharmaceutical, laboratory, and other inpatient services. The attending physicians recorded all abstract discharge data at discharge. Previous studies have reported the favorable sensitivity and specificity of the diagnoses and procedure records in the database.^21,22^ Furthermore, several perinatal studies have been conducted using this database.^23,24^

### Study protocol

We identified patients under 28 weeks of gestational age who were admitted to the NICUs between July 2010 and March 2021. We included patients who were transferred from other NICUs and non-NICU settings. We excluded patients who were admitted after 2 days of age and discharged before 2 days of age. Additionally, we excluded those who were diagnosed with trisomies 21, 18, and 13 (defined by the ICD-10 codes; Supplementary Table S1) and those who underwent cardiac surgeries other than the surgery for patent ductus arteriosus.

The original Japanese procedure codes were used to identify the cardiac surgeries. We categorized eligible patients into those who received opioids before 2 days of age (opioid group) and those who did not receive opioids (non-opioid group). We defined the exposure period as 2 days of age per previously established methodologies^24,25^ to evaluate the effects of early-phase opioid use because hemodynamics soon after birth can change drastically;^26^ thus, the effects of opioid use after 2 days of age were not assessed and the non-opioid group could receive opioids after 2 days of age. The period was defined by days instead of hours because the time of day for events was unavailable in the database. We consider the definition of 2 days appropriate because a single-day exposure period hardly allows a patient born at midnight to be classified into the exposure group.

The primary outcomes were in-hospital mortality, IVH, and PVL. The ICD-10 codes were used to define IVH and PVL (Supplementary Table S1).^24^ The grade of IVH and the status of in-utero IVH were unavailable because they were indistinguishable by ICD-10 codes. The secondary outcomes included other in-hospital morbidities (BPD, hydrocephalus, necrotizing enterocolitis, and retinopathy of prematurity), discharge destination, home assistive technology use (home oxygen therapy, tracheostomy, and home mechanical ventilation), length of hospital stay, length of intubation, and total hospitalization cost. BPD was defined as receiving oxygen, non-invasive positive-pressure ventilation, or invasive mechanical ventilation at 36 weeks of corrected gestational age. The ICD-10 codes were used to define hydrocephalus and necrotizing enterocolitis (Supplementary Table S1).^24^ We selected hydrocephalus as a secondary outcome as a proxy for severe IVH because we were unable to determine the actual grade of IVH. Retinopathy of prematurity was defined as receiving an antivascular endothelial growth factor injection or laser photocoagulation.^24^ We defined the currency exchange rate as 140 Japanese yen per 1 US dollar.

The following patient characteristics were investigated: sex, gestational age, birth weight, in-hospital birth, presence of neonatal asphyxia, and general comorbidities. Gestational age was categorized into three groups: 22–23, 24–25, and 26–27 weeks. Additionally, birth weight was categorized into four groups: <500, 500–999, 1000–1499, and ≥1500 g. The ICD-10 codes and original Japanese procedure codes were used to define neonatal asphyxia (Supplementary Table S1).^24^ The Pediatric Complex Chronic Conditions Classification System version 2 (CCC) was used to identify general comorbidities.^27^ CCC includes a variety of disease categories, such as cardiovascular diseases, genetic disorders, and neurological disorders. General comorbidities were categorized into four groups according to the number of CCC (0, 1, 2, and ≥3).

Subsequently, we investigated the following treatments before 2 days of age: drug use (adrenalin, dopamine or dobutamine, milrinone, cyclooxygenase inhibitors [ibuprofen or indomethacin], antibiotics, caffeine citrate, granulocyte-colony stimulating factor, steroids, muscle relaxants [rocuronium and vecuronium], and other sedative agents [midazolam, dexmedetomidine, and phenobarbital]), blood transfusion (albumin, fresh frozen plasma, gamma-globulin, platelets, and red blood cell), and procedures (arterial catheterization, central venous catheterization, inhaled nitric oxide, intratracheal surfactant, intubation, surgery for patent ductus arteriosus, and jaundice phototherapy). Additionally, we collected data on the hospital type (whether a patient was admitted to a general perinatal medical center or not, and to a teaching hospital or not), hospital volume, and fiscal year. NICUs in general perinatal medical centers in Japan are similar to the level 3 NICUs in the United States.^28^ Hospital volume was defined as the number of patients with gestational age < 28 weeks at each hospital and was categorized into three groups (low, medium, and high). The fiscal years were categorized into two groups: 2010–2015 and 2016–2021.

### Statistical analysis

To compare the outcomes between the two groups, we performed propensity score matching analysis at a 1:2 ratio.^29^ Propensity scores were calculated using a logistic regression model in which the independent variables included patient backgrounds and treatments. Each patient in the opioid group was matched with two patients with the closest estimated propensity scores within a caliper (≤ 0.2 of the pooled standard deviation) in the non-opioid group. The matched samples were obtained using the nearest-neighbor matching with replacement. Furthermore, we calculated the standardized differences to confirm the balance in baseline characteristics between the two groups for all patients, as well as for the 1:2 propensity score-matched cohort. A standardized difference of <10% implied a negligible difference between the two groups.^30^ We performed a χ^2^ test to compare proportions for categorical outcomes, and the Mann–Whitney U test to compare medians for continuous outcomes in the 1:2 propensity score-matched pair cohort. After generating the propensity score-matched pair cohort, we used a logistic regression model to calculate the odds ratio for the opioid group compared with the non-opioid group.

For sensitivity analysis, we conducted the following analyses to examine the robustness of our inference: overlap propensity score-weighted and instrumental variable analyses. Overlap propensity score-weighted analysis is an extension of the propensity score analysis that balances covariates between the two groups and imitates a randomized trial without excluding most observations from the study sample.^31^ In this analysis, we used the same propensity score as in the main analysis. Next, using a two-stage residual inclusion method, we conducted instrumental variable analysis. Instrumental variable analysis can theoretically adjust for the unmeasured background characteristics and resemble trials.^32–35^ We used the facility treatment rate as an instrumental variable because it is a typical instrumental variable in epidemiological studies.^33^ In the present study, the facility treatment rate was defined as the number of extremely preterm infants receiving opioids divided by the total number of eligible extremely preterm infants in each hospital. The validity of the instrumental variable was tested using its F-statistic; an F-statistic <10 was considered an invalid instrumental variable.^33^ A logistic regression model was used to calculate the odds ratio for the opioid group compared with the non-opioid group in each sensitivity analysis. Moreover, after generating the propensity score-matched pair cohort, we performed logistic regression analysis using the propensity score and all variables to calculate the odds ratio for the opioid group compared with the non-opioid group.

Finally, we performed subgroup analyses based on the gestational age and opioid type. We conducted a 1:2 propensity score-matching analysis in patients under 25 weeks of gestational age. This analysis included re-categorizing the birth weight into four groups (<500, 500–599, 600–699, and ≥700 g) for balancing the matched patients. Additionally, we further divided the opioid group into two groups (fentanyl and morphine groups) according to the opioid type used before 2 days of age and separately compared the outcomes with those of the non-opioid group. The subgroup analysis involved adjusting the matching ratio according to the number of patients in each group.

The significance threshold was set at 0.05, and all reported *p*-values were two-sided. All statistical analyses were conducted using Stata/MP 17.0 (StataCorp, College Station, TX).

## Results

**Fig. 1:**
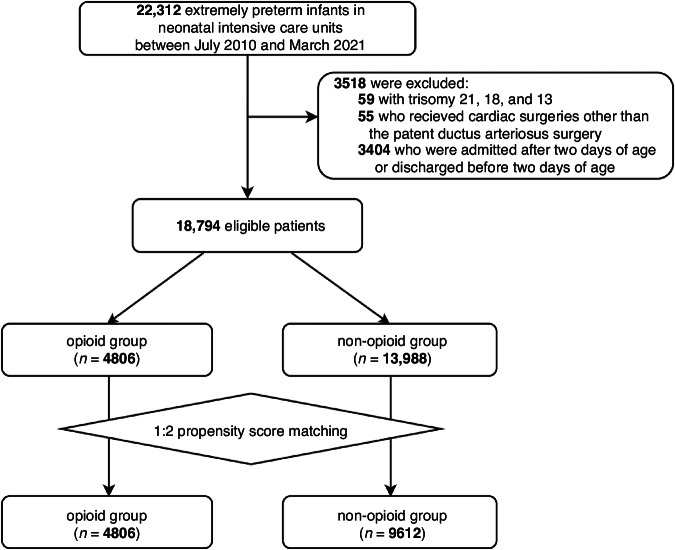
Patient flow. The non-opioid group comprised the patients who did not receive opioids within the first 2 days of age irrespective of opioids use after 2 days of age.

We identified 22,312 patients with a gestational age of <28 weeks who were treated in NICUs between July 2010 and March 2021. We excluded 3518 patients who met the following exclusion criteria: (i) 59 patients with trisomies 21, 18, and 13; (ii) 55 patients who underwent cardiac surgery other than the surgery for patent ductus arteriosus; and (iii) 3404 patients who were admitted or discharged after or before 2 days of age, respectively. Of the 18,794 eligible patients, the opioid and non-opioid groups comprised 4806 (26%) and 13,988 patients (74%), respectively (Fig. 1).

Table 1 summarizes the baseline characteristics of all patients and the patients with 1:2 propensity score matching. Before matching, the opioid group demonstrated a lower gestational age and birth weight and was more likely to receive almost all drugs, transfusions, and procedures than the non-opioid group. The non-opioid group comprised 9612 patients, and the background characteristics were well balanced between the two groups after the 1:2 propensity score matching.

**Table 1:** Background characteristics of all patients and patients with 1:2 propensity score matching.

	All patients					Patients with 1:2 propensity score matching				
	Opioid		Non-opioid^a^		ASD^b^	Opioid		Non-opioid^a^		ASD^b^
	*n* = 4806		*n* = 13,988		(%)	*n* = 4806		*n* = 9612		(%)
*Patient characteristics*										
Male	2556	(53)	7438	(53)	0.0	2556	(53)	5221	(54)	2.3
Gestational age category, weeks										
22–23	1178	(25)	1869	(13)	28.8	1178	(25)	2336	(24)	0.5
24–25	2099	(44)	4384	(31)	25.7	2099	(44)	4222	(44)	0.5
26–27	1529	(32)	7735	(55)	48.7	1529	(32)	3054	(32)	0.1
Birth weight category, g										
<500	711	(15)	1272	(9.1)	17.6	711	(15)	1493	(16)	2.1
500–999	3797	(79)	10,586	(76)	8.0	3797	(79)	7531	(78)	1.6
1000–1499	283	(5.9)	2053	(15)	29.2	283	(5.9)	566	(5.9)	0.0
≥1500	11	(0.23)	72	(0.51)	4.7	11	(0.23)	16	(0.17)	1.4
Missing data	4	(0.08)	5	(0.04)	1.9	4	(0.08)	6	(0.06)	0.8
In-hospital birth	4573	(95)	13,366	(96)	1.9	4573	(95)	9111	(95)	1.7
Number of CCC										
0	1456	(30)	4085	(29)	2.4	1456	(30)	2865	(30)	1.1
1	2939	(61)	8638	(62)	1.2	2939	(61)	5810	(60)	1.4
2	377	(7.8)	1148	(8.2)	1.3	377	(7.8)	863	(9.0)	4.1
≥3	34	(0.71)	117	(0.84)	1.5	34	(0.71)	74	(0.77)	0.7
Neonatal asphyxia	3838	(80)	11015	(79)	2.7	3838	(80)	7771	(81)	2.5
*Treatment characteristics*										
Drug use										
Adrenalin	85	(1.8)	155	(1.1)	5.5	85	(1.8)	145	(1.5)	2.0
Dopamine/Dobutamine	2491	(52)	5645	(40)	23.2	2491	(52)	5335	(56)	7.4
Milrinone	56	(1.2)	62	(0.44)	8.1	56	(1.2)	112	(1.2)	0.0
Cyclooxygenase inhibitors	1775	(37)	3894	(28)	19.5	1775	(37)	3600	(37)	1.1
Antibiotics	4309	(90)	9471	(68)	55.6	4309	(90)	8702	(91)	2.9
Antifungal agents	2181	(45)	2981	(21)	52.8	2181	(45)	4376	(46)	0.3
Caffeine citrate	83	(1.7)	621	(4.4)	15.7	83	(1.7)	182	(1.9)	1.2
G-CSF	86	(1.8)	222	(1.6)	1.6	86	(1.8)	187	(1.9)	1.2
Steroids	2648	(55)	2523	(18)	83.4	2648	(55)	5362	(56)	1.4
Muscle relaxants	40	(0.83)	12	(0.09)	11.1	40	(0.83)	55	(0.6)	3.1
Other sedative agents										
Dexmedetomidine	1	(0.02)	48	(0.34)	7.6	1	(0.02)	2	(0.02)	0.0
Midazolam	403	(8.4)	571	(4.1)	17.9	403	(8.4)	992	(10)	6.6
Pentazocine	2	(0.04)	2	(0.01)	1.6	2	(0.04)	3	(0.03)	0.5
Phenobarbital	1040	(22)	2564	(18)	8.3	1040	(22)	2451	(25)	9.1
Transfusion										
Albumin	476	(9.9)	1120	(8.0)	6.6	476	(9.9)	1099	(11)	5.0
Fresh frozen plasma	1093	(23)	1447	(10)	33.8	1093	(23)	2341	(24)	3.8
Gamma-globulin	2533	(53)	4410	(32)	43.9	2533	(53)	5021	(52)	0.9
Platelets	69	(1.4)	112	(0.8)	6.0	69	(1.4)	144	(1.5)	0.5
Red blood cell	626	(13)	672	(4.8)	29.2	626	(13)	1320	(14)	2.1
Procedures										
Arterial catheterization	2772	(58)	4663	(33)	50.4	2772	(58)	5709	(59)	3.5
Central venous catheterization	3950	(82)	9835	(70)	28.2	3950	(82)	8092	(84)	5.3
Inhaled nitric oxide	408	(8.5)	335	(2.4)	27.1	408	(8.5)	839	(8.7)	0.9
Intratracheal surfactant	3968	(83)	9000	(64)	42.2	3968	(83)	7972	(83)	1.0
Intubation	3956	(82)	9717	(70)	30.4	3956	(82)	8259	(86)	9.9
Phototherapy	891	(19)	2503	(18)	1.7	891	(19)	1851	(19)	1.8
Teaching hospital	4735	(99)	11,880	(85)	50.9	4735	(99)	9549	(99)	8.0
General Perinatal Medical Center	4649	(97)	13,494	(96)	1.5	4649	(97)	9325	(97)	1.6
Hospital volume										
<12	1588	(33)	4349	(31)	4.2	1588	(33)	3362	(35)	4.1
12–22	1819	(38)	4607	(33)	10.3	1819	(38)	3296	(34)	7.4
>22	1399	(29)	5032	(36)	14.7	1399	(29)	2954	(31)	3.5
Fiscal year of surgery										
2010–2015	2015	(42)	7231	(52)	19.7	2015	(42)	4054	(42)	0.5
2016–2021	2791	(58)	6757	(48)	19.7	2791	(58)	5558	(58)	0.5

*ASD* Absolute standardized difference, *CCC* Complex chronic condition (pediatric complex chronic conditions classification system, version 2), *G-CSF* granulocyte colony-stimulating factor.

^a^The non-opioid group comprised the patients who did not receive opioids within the first 2 days of age irrespective of opioids use after 2 days of age. ^b^An ASD of ≤ 10% denotes a negligible difference between the two groups.

**Fig. 2:**
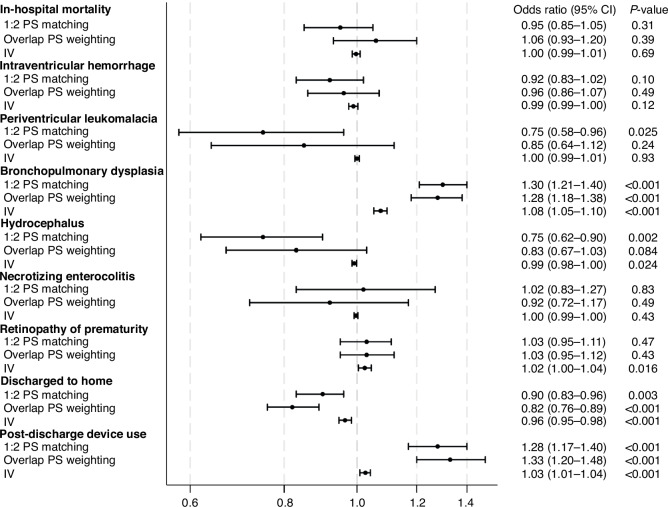
Odds ratios for the opioid versus the non-opioid groups for each outcome in the propensity score and instrumental variable analyses. Odds ratios are with reference to patients in the non-opioid group. The non-opioid group comprised the patients who did not receive opioids within the first 2 days of age irrespective of opioids use after 2 days of age. CI confidence interval, IV instrumental variable analysis, PS propensity score.

Table 2 demonstrates the crude outcomes of all patients and the 1:2 propensity score-matched cohort. Among all patients, the in-hospital mortality was 8.5%, and IVH and PVL occurred in 2365 (13%) and 388 (2.1%) patients, respectively. The in-hospital mortality and IVH did not differ between the two groups after the 1:2 propensity score matching. The opioid group exhibited significantly lower PVL (1.7% vs. 2.2%), hydrocephalus (3.3% vs. 4.3%), and home discharge occurrences (66% vs. 68%) but higher BPD (65% vs. 58%) and home assistive technology use occurrences (19% vs. 15%) than the non-opioid group. Moreover, the lengths of hospital stay (125 vs. 122 days) and intubation (73 vs. 71 days) were longer, and total hospitalization costs (89,485 vs. 86,827 US dollars) were higher in the opioid group than in the non-opioid group. Similar results were observed in the logistic regression analysis after generating the propensity score-matched pair cohort (Fig. 2).

**Table 2:** Comparisons of the outcomes between the opioid and non-opioid groups among all patients and patients with 1:2 propensity score matching.

	All patients					Patients with 1:2 propensity score matching				
	Opioid		Non-opioid^a^			Opioid		Non-opioid^a^		
	*n* = 4806		*n* = 13,988		*p*-value	*n* = 4806		*n* = 9612		*p*-value
In-hospital mortality	571	(12)	1,022	(7.3)	<0.001	571	(12)	1199	(12)	0.31
Intraventricular hemorrhage	692	(14)	1,672	(12)	<0.001	692	(14)	1485	(15)	0.10
Periventricular leukomalacia	81	(1.7)	307	(2.2)	0.032	81	(1.7)	216	(2.2)	0.025
Other in-hospital morbidities										
Bronchopulmonary dysplasia	3103	(65)	7347	(53)	<0.001	3103	(65)	5574	(58)	<0.001
Hydrocephalus	158	(3.3)	371	(2.7)	0.022	158	(3.3)	418	(4.3)	0.002
Necrotizing enterocolitis	128	(2.7)	298	(2.1)	0.032	128	(2.7)	250	(2.6)	0.83
Retinopathy of prematurity	1573	(33)	3762	(27)	<0.001	1573	(33)	3088	(32)	0.47
Discharged to home	3148	(66)	10,533	(75)	<0.001	3148	(66)	6530	(68)	0.003
Post-discharge device use	898	(19)	1684	(12)	<0.001	898	(19)	1463	(15)	<0.001

	Median	IQR	Median	IQR		Median	IQR	Median	IQR	
Length of hospital stay, days	125	(97–153)	114	(92–142)	<0.001	125	(97–153)	122	(93–153)	0.002
Length of intubation, days	73	(47–97)	59	(37–82)	<0.001	73	(47–97)	71	(43–93)	<0.001
Total hospitalization cost, US dollars	89,485	(70,263–104,956)	77,756	(59,098–94,748)	<0.001	89,485	(70,263–104,956)	86,827	(66,018–102,865)	<0.001

*IQR* interquartile range.

^a^The non-opioid group comprised the patients who did not receive opioids within the first 2 days of age irrespective of opioids use after 2 days of age.

The weighted background characteristics revealed an exact balance between the two groups in the overlap propensity score-weighted analysis (Supplementary Table S2) and the results corresponded with the main analysis (Supplementary Table S3). Additionally, the instrumental variable analysis had 1310 as the F-statistic and similar results to the propensity score analyses (Fig. 2). Similar results were observed in the logistic regression analysis using the propensity score and all variables after generating the propensity score-matched pair cohort (Supplementary Table S4).

The subgroup analyses’ background characteristics after propensity score matching were well-balanced (Supplementary Tables S5–6). The in-hospital mortality and morbidity rates of patients <25 weeks of gestational age were higher than those of all patients (Table 3). Although mortality did not differ between the two groups in line with the main analysis, opioid use was associated with decreased IVH (17% vs. 21%). Regarding the opioid type, the fentanyl group demonstrated results similar to those of the main analysis (e.g., decreased occurrence of PVL); however, the morphine group was less likely to develop IVH than the non-opioid group (13% vs. 17%) (Table 4).

**Table 3:** Comparisons of the outcomes between the opioid and non-opioid groups among the patients < 25 weeks of gestational age and patients with 1:1 propensity score matching.

	Patients < 25 weeks of Gestational age					Patients with 1:1 propensity score matching				
	Opioid		Non-opioid^a^			Opioid		Non-opioid^a^		
	*n* = 2259		*n* = 3744		*p*-value	*n* = 2256		*n* = 2256		*p*-value
In-hospital mortality	398	(18)	605	(16)	0.14	398	(18)	383	(17)	0.56
Intraventricular hemorrhage	378	(17)	686	(18)	0.12	378	(17)	464	(21)	0.001
Periventricular leukomalacia	28	(1.2)	62	(1.7)	0.20	28	(1.2)	42	(1.9)	0.092
Other in-hospital morbidities										
Bronchopulmonary dysplasia	1544	(68)	2350	(63)	<0.001	1542	(68)	1415	(63)	<0.001
Hydrocephalus	91	(4.0)	165	(4.4)	0.48	91	(4.0)	132	(5.9)	0.005
Necrotizing enterocolitis	76	(3.4)	137	(3.7)	0.55	76	(3.4)	98	(4.3)	0.089
Retinopathy of prematurity	890	(39)	1517	(41)	0.39	888	(39)	949	(42)	0.065
Discharged to home	1332	(59)	2446	(65)	<0.001	1329	(59)	1409	(62)	0.015
Home assistive technology use	522	(23)	726	(19)	0.001	520	(23)	407	(18)	<0.001

	Median	IQR	Median	IQR		Median	IQR	Median	IQR	
Length of hospital stay, days	139	(107–168)	140	(111–170)	0.16	139	(107.5–168)	138	(107–169)	0.87
Length of intubation, days	86	(58–111)	80	(53–102)	<0.001	86	(58–111)	85	(57–108)	0.18
Total hospitalization cost, US dollars	98,252	(79,389–113,339)	94,208	(70,475–109,023)	<0.001	98,294	(79,350–113,341)	96,986	(75,502–112,524)	0.12

*IQR* interquartile range.

^a^The non-opioid group comprised the patients who did not receive opioids within the first 2 days of age irrespective of opioids use after 2 days of age.

**Table 4:** Comparisons of the outcomes between the fentanyl group or the morphine group and non-opioid group among patients and patients with propensity score matching.

	Patients with 1:3 propensity score matching without morphine					Patients with 1:4 propensity score matching without fentanyl				
	Fentanyl		Non-opioid^a^			Morphine		Non-opioid^a^		
	*n* = 3808		*n* = 11,245		*p*-value	*n* = 997		*n* = 3988		*p*-value
In-hospital mortality	457	(12)	1378	(12)	0.92	115	(12)	517	(13)	0.23
Intraventricular hemorrhage	565	(15)	1641	(14)	0.47	129	(13)	670	(17)	0.003
Periventricular leukomalacia	63	(1.7)	265	(2.3)	0.014	19	(1.9)	89	(2.2)	0.53
Other in-hospital morbidities										
Bronchopulmonary dysplasia	2450	(64)	6639	(58)	<0.001	651	(65)	2229	(56)	<0.001
Hydrocephalus	139	(3.7)	435	(3.8)	0.66	19	(1.9)	155	(3.9)	0.002
Necrotizing enterocolitis	97	(2.5)	303	(2.7)	0.73	31	(3.1)	83	(2.1)	0.052
Retinopathy of prematurity	1191	(31)	3766	(33)	0.054	382	(38)	1240	(31)	<0.001
Discharged to home	2477	(65)	7778	(68)	0.001	670	(67)	2680	(67)	0.99
Post-discharge device use	696	(18)	1651	(14)	<0.001	201	(20)	554	(14)	<0.001

	Median	IQR	Median	IQR		Median	IQR	Median	IQR	
Length of hospital stay, days	125	(97–154)	121	(94–151)	<0.001	124	(95–149)	120	(93–152)	0.19
Length of intubation, days	72	(46–97)	70	(44–91)	<0.001	74	(48–96)	67	(41–93)	<0.001
Total hospitalization cost, US dollars	90,327	(70,960–105,181)	86,856	(66,902–102,262)	<0.001	86,052	(67,137–103,638)	84,919	(64,189–101,082)	0.023

*IQR* interquartile range.

^a^The non-opioid group comprised the patients who did not receive opioids within the first 2 days of age irrespective of opioids use after 2 days of age.

## Discussion

This study investigated the relationship between early-phase opioid use and outcomes in extremely preterm infants using a nationwide database in Japan. Previous studies reported higher mortality (odds ratio 1.57 [95% confidence interval, 1.13–2.18]) and IVH (1.63 [1.30–2.04]) in opioid users;^7,17^ however, after adjusting for background factors, our analysis found no significant differences in mortality or IVH between the opioid and non-opioid groups. Additionally, this study revealed that early-phase opioid use was linked to decreased PVL, but also increased BPD, length of hospital stay, and hospitalization costs.

Preventing IVH is a critical goal in perinatal medicine as it is a leading cause of neurodevelopmental impairment in extremely preterm infants.^3,5^ A previous observational study with inadequate adjustment for confounders found that opioid use in extremely preterm infants was linked to increased in-hospital mortality and IVH.^7^ In the current study, after adjusting for various clinical backgrounds and treatments, opioid use was not linked to IVH but was associated with a reduced incidence of hydrocephalus. This reflects a decrease in severe IVH in the opioid group as hydrocephalus usually develops with severe IVH.^36^ In other words, early-phase opioid use may reduce IVH severity in extremely preterm infants.

Patients in the opioid group were more likely to exhibit BPD than those in the non-opioid group. Opioid use may extend intubation duration as previously reported,^1^ and lead to BPD from ventilator-associated lung damage in extremely preterm infants.^37^

Long-term intubation may also lead to increased home assistive technology use, longer hospital stays, and higher hospitalization costs in the opioid group. Clinicians should avoid long intubation duration when using early-phase opioids in extremely preterm infants. This study showed that early-phase opioid use was significantly associated with an increase in the occurrence of BPD, with only a slight decrease in the occurrence of PVL. Therefore, clinicians should consider limiting routine early-phase opioid use until further data are available from well-designed and adequately powered prospective cohort studies.

Extremely preterm infants born <25 weeks of gestation are at a particularly high risk for developing IVH.^38,39^ However, limited evidence exists regarding the effectiveness of early-phase opioid use in these infants. The present study involving >6000 extremely preterm infants showed that early-phase opioid use at <25 weeks of gestational age was associated with reduced occurrence of IVH and hydrocephalus. Preventing IVH is crucial as it is an important risk factor for developing neurodevelopmental impairment.^3^ Nevertheless, early-phase opioids markedly increased BPD in this population. Clinicians should avoid routine early-phase opioid use without considering the increased BPD risk, even in this population.

Although morphine is a major analgesic opioid, fentanyl is often used as an alternative due to its fewer cardiovascular and gastrointestinal side effects than morphine^40^; indeed, fentanyl was used approximately four times more frequently than morphine in this study. However, fentanyl was not linked to decreased IVH, whereas morphine was associated with reduced IVH. Fentanyl might have a weak analgesic effect in preventing IVH. Additionally, the fentanyl group (as well as morphine groups) exhibited a higher BPD incidence, longer hospital stays and intubation duration, and increased hospitalization costs than the non-opioid group. Fentanyl should be carefully selected as an early-phase opioid to reduce side effects as its use demonstrated no benefit (other than a small reduction in PVL).

This study has several limitations. First, we could not obtain information on the drug dosage and administration methods, such as continuous or bolus administration. Therefore, this study did not examine the dose-dependent effects of early-phase opioid use. Second, we lacked access to laboratory data (e.g., blood gas tests) and imaging findings (e.g., magnetic resonance imaging and echocardiography) to assess the severity of patient backgrounds and outcomes. We could not determine the severity of IVH and the actual onset of IVH in this study. However, even in clinical practice, it is difficult to determine the actual onset of IVH by ultrasound, which is commonly used for the diagnosis of IVH in neonates. Information on maternal history, including antenatal corticosteroid administration, delivery mode, and maternal comorbidities, was not obtained. However, we adjusted for neonatal conditions after birth, including patient background, drugs, and procedures, through propensity scores and instrumental variable analyses. We believe these adjustments can account for measured and unmeasured covariates. Fourth, we did not assess the effects of opioid use beyond 2 days of age because we originally aimed to evaluate the effects of early-phase opioid use. Finally, we did not assess the long-term effects of early-phase opioid use in extremely preterm infants. However, our study focused on the short-term effects of early-phase opioid use in extremely preterm infants because data on these is limited, even in short-term outcomes.

## Conclusions

We performed propensity score and instrumental variable analyses with a large nationwide cohort of extremely preterm infants born under 28 weeks of gestation to compare the short-term effects of early-phase opioid use. Early-phase opioids for extremely preterm infants were markedly associated with decreased PVL and hydrocephalus, increased BPD, prolonged length of hospital stay and intubation, and higher hospitalization costs. Furthermore, Early phase opioid use was not considerably linked to increased IVH incidence.

## Supplementary information

Supplementary_file

## Data Availability

The data analyzed in this study are not publicly available due to contracts with the hospitals providing data to the database. Further inquiries can be directed to the corresponding author.
