# Supplementary material for: Association between early-phase opioid use and outcomes in extremely preterm infants: A nationwide study
Source: Pediatr Res. 2025 Mar 18;98(5):1841–8. doi: 10.1038/s41390-025-03998-y (PMC12602305; doi:10.1038/s41390-025-03998-y)
Supplement: Supplementary file 1 — Supplementary_file [file 41390_2025_3998_MOESM1_ESM.pdf]

Association between early-phase opioid use and outcomes in extremely preterm infants: A nationwide database study

Hiroki Kitaoka, Takaaki Konishi, Yoshihiko Shitara, Atsushi Ito, Kohei Kashima, Yuya Kimura, Hiroki Matsui, Motohiro Kato, Naoto Takahashi, and Hideo Yasunaga

Supplementary Table S1. ICD-10 code definitions
Supplementary Table S2. Background characteristics of patients with overlapping propensity score weighting
Supplementary Table S3. Overlapping propensity score-weighted and instrumental variable analyses were used to compare the opioid and non-opioid groups among all patients.
Supplementary Table S4. Logistic regression analysis using the propensity score and all variables after generating the propensity score-matched pair cohort for all patients
Supplementary Table S5. Background characteristics of patients with < 25 weeks of gestational age and patients with 1:1 propensity score matching.
Supplementary Table S6. Background characteristics of the patients with propensity score matching in the fentanyl, morphine, and non-opioid groups.

Supplementary Table S1. ICD-10 code definitions

Comorbidity	ICD-10 codes
Hydrocephalus	G91, Q039
Intraventricular hemorrhage	I60, I61, I62, P52
Necrotizing enterocolitis	A052, P77
Neonatal asphyxia	P21
Periventricular leukomalacia	P912
Trisomy 13	Q914, Q915, Q916, Q917
Trisomy 18	Q910, Q911, Q912, Q913
Trisomy 21	Q90

Abbreviation: ICD, International Classification of Diseases

Supplementary Table S2. Background characteristics of all patients with overlap propensity scores weighting

	Proportions with overlap propensity-score weighting	
	All patients	
	Opioid	Non-opioid*
Patient characteristics		
Male	(53)	(53)
Gestational age category, weeks		
22–23	(21)	(21)
24–25	(41)	(41)
26–27	(38)	(38)
Birth weight category, g		
<500	(13)	(13)
500–999	(79)	(79)
1000–1499	(7.6)	(7.6)
≥1500	(0.24)	(0.24)
Missing data	(0.07)	(0.07)
In-hospital birth	(95)	(95)
Number of CCC		
0	(29)	(29)
1	(62)	(62)
2	(8.0)	(8.0)
≥3	(0.74)	(0.74)
Neonatal asphyxia	(80)	(80)
Treatment characteristics		
Drug use		
Adrenalin	(47)	(47)
Dopamine/Dobutamine	(52)	(52)
Milrinone	(0.91)	(0.91)
Cyclooxygenase inhibitors	(7.4)	(7.4)
Antibiotics	(86)	(86)
Antifungal agents	(38)	(38)
Caffeine citrate	(2.2)	(2.2)
G-CSF	(1.9)	(1.9)
Steroids	(42)	(42)
Muscle relaxants	(0.32)	(0.32)
Other sedative agents		
Dexmedetomidine	(0.04)	(0.04)
Midazolam	(7.7)	(7.7)
Pentazocine	(0.02)	(0.02)
Phenobarbital	(24)	(24)
Transfusion		
Albumin	(10)	(10)
Fresh frozen plasma	(20)	(20)
Gamma-globulin	(47)	(47)
Platelets	(1.3)	(1.3)
Red blood cell	(10)	(10)
Procedures		
Arterial catheterization	(52)	(52)
Central venous catheterization	(82)	(82)
Inhaled nitric oxide	(5.8)	(5.8)
Intratracheal surfactant	(81)	(81)
Intubation	(83)	(83)
Phototherapy	(20)	(20)
Teaching hospital	(98)	(98)
General perinatal medical center	(97)	(97)
Hospital volume		
<12	(33)	(33)
12–22	(35)	(35)
>22	(32)	(32)
Fiscal year of surgery		
2010–2015	(46)	(46)
2016–2021	(54)	(54)

Data are presented as numbers (%). **Abbreviations**: CCC, Complex chronic condition (pediatric complex chronic conditions classification system, version 2); G-CSF, Granulocyte Colony Stimulating Factor. *The non-opioid group comprised the patients who did not receive opioids within the first 2 days of age irrespective of opioids use after 2 days of age.

Supplementary Table S3. Overlapping propensity score-weighted and instrumental variable analyses were used to compare the opioid and non-opioid groups among all patients.

	Overlap propensity score weighted analysis			Instrumental variable analysis*		
	OR†	95% CI	p-value	OR†	95% CI	p-value
In-hospital mortality	1.06	(0.93–1.20)	0.39	1.00	(0.99–1.01)	0.69
Intraventricular hemorrhage	0.96	(0.86–1.07)	0.49	0.99	(0.99–1.00)	0.12
Periventricular leukomalacia	0.85	(0.64–1.12)	0.24	1.00	(0.99–1.01)	0.93
Other in-hospital morbidities						
Bronchopulmonary dysplasia	1.28	(1.18–1.38)	<0.001	1.08	(1.05–1.10)	<0.001
Hydrocephalus	0.83	(0.67–1.03)	0.084	0.99	(0.98–1.00)	0.024
Necrotizing enterocolitis	0.92	(0.72–1.17)	0.49	1.00	(0.99–1.00)	0.43
Retinopathy of prematurity	1.03	(0.95–1.12)	0.43	1.02	(1.00–1.04)	0.016
Discharged to home	0.82	(0.76–0.89)	<0.001	0.96	(0.95–0.98)	<0.001
Post-discharge device use	1.33	(1.20–1.48)	<0.001	1.03	(1.01–1.04)	0.001
	Diff†	95% CI	p-value	Diff†	95% CI	p-value
Length of hospital stay, days	0.63	(-2.61–3.87)	0.70	2.7	(-0.81–6.17)	0.13
Length of intubation, days	3.36	(0.67–6.05)	0.014	4.1	(1.18–7.10)	0.006
Total hospitalization cost, US dollars	2167	(636–3698)	0.006	3339	(1,712–4,966)	<0.001

Abbreviations: OR, odds ratio; CI, confidence interval; Diff, difference

*The F-statistic was 1310.

†Odds ratios and differences are with reference to patients in the non-opioid group. The non-opioid group comprised the patients who did not receive opioids within the first 2 days of age irrespective of opioids use after 2 days of age.

Supplementary Table S4. Logistic regression analysis using the propensity score and all variables after generating the propensity score-matched pair cohort for all patients

	All patients		
	OR†	95% CI	p-value
In-hospital mortality	0.97	(0.81–1.17)	0.78
Intraventricular hemorrhage	0.93	(0.74–1.16)	0.52
Periventricular leukomalacia	0.70	(0.51–1.25)	0.32
Other in-hospital morbidities			
Bronchopulmonary dysplasia	1.33	(1.15–1.55)	<0.001
Hydrocephalus	0.83	(0.63–1.05)	0.11
Necrotizing enterocolitis	1.03	(0.75–1.14)	0.84
Retinopathy of prematurity	1.05	(0.86–1.30)	0.63
Discharged to home	0.88	(0.76–1.03)	0.10
Post-discharge device use	1.32	(1.11–1.55)	0.001

Abbreviations: OR, odds ratio; CI, confidence interval

†Odds ratios are with reference to patients in the non-opioid group. The non-opioid group comprised the patients who did not receive opioids within the first 2 days of age irrespective of opioids use after 2 days of age.

Supplementary Table S5. Background characteristics of patients with < 25 weeks of gestational age and patients with 1:1 propensity score matching.

	Patients <25 weeks of GA			1:1 Propensity score-matched patients		
	Opioid *n*=2259	Non-opioid* *n*=3744	ASD† (%)	Opioid *n*=2256	Non-opioid* *n*=2256	ASD† (%)
Patient characteristics						
Male	1196 (53)	2026 (54)	2.3	1196 (53)	1175 (52)	1.9
Gestational age category, weeks						
22	286 (13)	465 (12)	0.7	285 (13)	280 (12)	0.7
23	892 (39)	1404 (38)	4.1	891 (39)	842 (37)	4.5
24	1081 (48)	1875 (50)	4.5	1080 (48)	1134 (50)	4.8
Birth weight category, g						
<500	505 (22)	775 (21)	4.0	504 (22)	575 (25)	7.4
500–599	758 (34)	1,192 (32)	3.7	758 (34)	692 (31)	6.3
600–699	659 (29)	1,192 (32)	5.8	658 (29)	663 (29)	0.5
≥700	336 (15)	582 (16)	1.9	335 (15)	325 (14)	1.3
Missing data	1 (0.04)	3 (0.08)	1.4	1 (0.04)	1 (0.04)	0.0
In-hospital birth	2158 (96)	3591 (96)	1.9	2155 (96)	2160 (96)	1.1
Number of CCC						
0	588 (26)	825 (22)	9.4	586 (26)	532 (24)	5.5
1	1454 (64)	2469 (66)	3.3	1453 (64)	1,489 (66)	3.4
2	198 (8.8)	417 (11)	7.9	198 (08.8)	222 (10)	3.7
≥3	19 (0.84)	33 (0.88)	0.4	19 (0.84)	13 (0.58)	3.2
Neonatal asphyxia	1871 (83)	3152 (84)	3.7	1869 (83)	1865 (83)	0.5
Treatment characteristics						
Drug use						
Adrenalin	57 (2.5)	90 (2.4)	0.8	56 (2.5)	61 (2.7)	1.4
Dopamine/Dobutamine	1297 (57)	1724 (46)	22.9	1294 (57)	1376 (61)	7.4
Milrinone	29 (1.3)	20 (0.5)	7.9	29 (1.3)	32 (1.4)	1.2
Cyclooxygenase inhibitors	983 (44)	1340 (36)	15.8	982 (44)	992 (44)	0.9
Antibiotics	2085 (92)	2635 (70)	58.6	2082 (92)	2097 (93)	2.5
Antifungal agents	1205 (53)	1306 (35)	37.8	1203 (53)	1286 (57)	7.4
Caffeine citrate	34 (1.5)	49 (1.3)	1.7	34 (1.5)	43 (1.9)	3.1
G-CSF	37 (1.6)	55 (1.5)	1.4	37 (1.6)	39 (1.7)	0.7
Steroids	1377 (61)	989 (26)	74.3	1375 (61)	1376 (61)	0.1
Muscle relaxants	12 (0.53)	6 (0.16)	6.3	11 (0.49)	6 (0.27)	3.6
Other sedative agents						
Dexmedetomidine	0 (0.0)	16 (0.43)	9.3	0 (0.0)	0 (0.0)	NA
Midazolam	170 (7.5)	179 (4.8)	11.4	169 (7.5)	158 (7.0)	1.9
Pentazocine	2 (0.09)	0 (0.0)	4.2	0 (0.0)	0 (0.0)	NA
Phenobarbital	537 (24)	946 (25)	3.5	536 (24)	615 (27)	8.0
Transfusion						
Albumin	247 (11)	366 (9.8)	3.8	246 (11)	279 (12)	4.6
Fresh frozen plasma	631 (28)	702 (19)	21.8	629 (28)	653 (29)	2.4
Gamma-globulin	1346 (60)	1553 (41)	36.8	1343 (60)	1341 (59)	0.2
Platelets	30 (1.3)	48 (1.3)	0.4	30 (1.3)	29 (1.3)	0.4
Red blood cell	393 (17)	370 (9.9)	22.0	391 (17)	400 (18)	1.0
Procedures						
Arterial catheterization	1288 (57)	1459 (39)	36.7	1285 (57)	1260 (56)	2.2
Central venous catheterization	1813 (80)	2475 (66)	32.4	1810 (80)	1851 (82)	4.6
Inhaled nitric oxide	233 (10)	163 (4.4)	23.0	230 (10)	297 (13)	9.3
Intratracheal surfactant	1909 (85)	2496 (67)	42.5	1907 (85)	1946 (86)	4.9
Intubation	1829 (81)	2604 (70)	26.7	1827 (81)	1911 (85)	9.9
Phototherapy	447 (20)	683 (18)	3.9	447 (20)	473 (21)	2.9
Teaching hospital	2219 (98)	3098 (83)	54.7	2216 (98)	2234 (99)	6.9
General perinatal medical center	2192 (97)	3683 (98)	8.9	2189 (97)	2196 (97)	1.9
Hospital volume						
<4	825 (37)	1176 (31)	10.8	824 (37)	859 (38)	3.2
4–7	763 (34)	1215 (32)	2.8	761 (34)	667 (30)	9.0
>7	671 (30)	1353 (36)	13.7	671 (30)	730 (32)	5.7
Fiscal year of surgery						
2010–2015	942 (42)	1952 (52)	21.0	941 (42)	923 (41)	1.6
2016–2021	1317 (58)	1792 (48)	21.0	1315 (58)	1333 (59)	1.6

Abbreviations: ASD, Absolute standardized difference; CCC, Complex chronic condition (pediatric complex chronic conditions classification system, version 2); GA, Gestational age; G-CSF, Granulocyte Colony Stimulating Factor * The non-opioid group

comprised the patients who did not receive opioids within the first 2 days of age irrespective of opioids use after 2 days of age.
†An ASD of ≤10% denotes a negligible difference between the two group .

Supplementary Table S6. Background characteristics of the patients with propensity score matching in the fentanyl, morphine, and non-opioid groups.

	1:3 propensity score-matched patients					1:4 Propensity score-matched patients				
	Fentanyl *n*=3808		Non-opioid* *n*=11,425		ASD† (%)	Morphine *n*=997		Non-opioid* *n*=3988		ASD† (%)
Patient characteristics										
Male	2019	(53)	6105	(53)	0.8	538	(54)	2183	(55)	1.6
Gestational age category, weeks										
22–23	942	(25)	2860	(25)	0.7	234	(23)	887	(22)	2.9
24–25	1627	(43)	4863	(43)	0.3	470	(47)	1915	(48)	1.8
26–27	1239	(33)	3702	(32)	0.3	293	(29)	1186	(30)	0.8
Birth weight category, g										
<500	566	(15)	1872	(16)	4.2	143	(14)	605	(15)	2.3
500–999	3005	(79)	8896	(78)	2.5	790	(79)	3085	(77)	4.6
1000–1499	224	(5.9)	640	(5.6)	1.2	62	(6.2)	287	(7.2)	3.9
≥1500	10	(0.50)	12	(0.20)	5.1	1	(0.19)	7	(0.32)	2.7
Missing data	3	(0.08)	5	(0.04)	1.4	1	(0.10)	4	(0.10)	0.0
In-hospital birth	3634	(95)	10,898	(95)	0.2	938	(94)	3717	(93)	3.6
Number of CCC										
0	1061	(28)	3156	(28)	0.5	391	(39)	1614	(40)	2.6
1	2416	(63)	7253	(63)	0.1	525	(53)	2090	(52)	0.5
2	302	(7.9)	934	(8.2)	0.9	75	(07.5)	261	(6.5)	3.8
≥3	29	(0.76)	82	(0.72)	0.5	6	(0.60)	23	(0.58)	0.3
Neonatal asphyxia	3226	(85)	9718	(85)	1.0	614	(62)	2429	(61)	1.4
Treatment characteristics										
Drug use										
Adrenalin	69	(1.8)	216	(1.9)	0.6	16	(1.6)	61	(1.5)	0.6
Dopamine/Dobutamine	1894	(50)	6143	(54)	8.1	595	(60)	2433	(61)	2.7
Milrinone	43	(1.1)	140	(1.2)	0.9	13	(1.3)	35	(0.88)	4.1
Cyclooxygenase inhibitors	1606	(42)	4919	(43)	1.8	171	(17)	666	(17)	1.2
Antibiotics	3466	(91)	10528	(92)	4.1	840	(84)	3315	(83)	3.1
Antifungal agents	1781	(47)	5548	(49)	3.6	399	(40)	1614	(40)	0.9
Caffeine citrate	59	(1.5)	209	(1.8)	2.2	24	(2.4)	105	(2.6)	1.4
G-CSF	75	(2.0)	227	(2.0)	0.1	11	(1.1)	45	(1.1)	0.2
Steroids	2211	(58)	6,558	(57)	1.3	435	(44)	1742	(44)	0.1
Muscle relaxants	37	(1.0)	97	(0.85)	1.3	3	(0.30)	13	(0.33)	0.4
Other sedative agents										
Dexmedetomidine	1	(0.03)	5	(0.04)	0.9	0	(0.0)	0	(0.0)	NA
Midazolam	365	(9.6)	1276	(11)	5.2	39	(3.9)	153	(3.8)	0.4
Pentazocine	2	(0.05)	0	(0.0)	3.2	0	(0.0)	0	(0.0)	NA
Phenobarbital	862	(23)	3035	(27)	9.1	180	(18)	753	(19)	2.1
Transfusion										
Albumin	396	(10)	1278	(11)	2.5	81	(8.1)	348	(8.7)	2.2
Fresh frozen plasma	908	(24)	2923	(26)	4.0	183	(18)	759	(19)	1.7
Gamma-globulin	2076	(55)	6093	(53)	2.4	458	(46)	1809	(45)	1.2
Platelets	57	(1.5)	189	(1.7)	1.3	12	(1.2)	44	(1.1)	0.9
Red blood cell	512	(13)	1,544	(14)	0.2	114	(11)	484	(12)	2.2
Procedures										
Arterial catheterization	2265	(59)	6937	(61)	2.5	506	(51)	1996	(50)	1.4
Central venous catheterization	3167	(83)	9784	(86)	6.8	782	(78)	3112	(78)	1.0
Inhaled nitric oxide	319	(8.4)	954	(8.4)	0.1	87	(8.7)	439	(11)	7.7
Intratracheal surfactant	3158	(83)	9577	(84)	2.4	808	(81)	3204	(80)	1.8
Intubation	3112	(82)	9753	(85)	9.8	843	(85)	3413	(86)	2.9
Phototherapy	743	(20)	2255	(20)	0.6	147	(15)	565	(14)	1.6
Teaching hospital	3746	(98)	11,338	(99)	8.0	988	(99)	3963	(99)	3.2
General perinatal medical center	3694	(97)	11,063	(97)	1.0	954	(96)	3830	(96)	1.8
Hospital volume										
<12	1264	(33)	4084	(36)	5.4	326	(33)	1269	(32)	1.9
12–22	1302	(34)	3545	(31)	6.8	515	(52)	2011	(50)	2.5
>22	1242	(33)	3796	(33)	1.3	156	(16)	708	(18)	5.6
Fiscal year of surgery										
2010–2015	1486	(39)	4523	(40)	1.2	526	(53)	1968	(49)	6.8
2016–2021	2322	(61)	6902	(60)	1.2	471	(47)	2020	(51)	6.8

Abbreviations: ASD, Absolute standardised difference; CCC, Complex chronic condition (pediatric complex chronic conditions classification system, version 2); G-CSF, Granulocyte Colony Stimulating Factor

[*]The non-opioid group comprised the patients who did not receive opioids within the first 2 days of age irrespective of opioids use after 2 days of age. [†]An ASD of ≤10% denotes a negligible difference between the two groups.
